# Tyrosine Kinase Receptor Landscape in Lung Cancer: Therapeutical Implications

**DOI:** 10.1155/2016/9214056

**Published:** 2016-07-26

**Authors:** A. Quintanal-Villalonga, Luis Paz-Ares, Irene Ferrer, S. Molina-Pinelo

**Affiliations:** ^1^Medical Oncology Department, Hospital Universitario Doce de Octubre, 28041 Madrid, Spain; ^2^Centro Nacional de Investigaciones Oncológicas (CNIO), 28029 Mardid, Spain

## Abstract

Lung cancer is a heterogeneous disease responsible for the most cases of cancer-related deaths. The majority of patients are clinically diagnosed at advanced stages, with a poor survival rate. For this reason, the identification of oncodrivers and novel biomarkers is decisive for the future clinical management of this pathology. The rise of high throughput technologies popularly referred to as “omics” has accelerated the discovery of new biomarkers and drivers for this pathology. Within them, tyrosine kinase receptors (TKRs) have proven to be of importance as diagnostic, prognostic, and predictive tools and, due to their molecular nature, as therapeutic targets. Along this review, the role of TKRs in the different lung cancer histologies, research on improvement of anti-TKR therapy, and the current approaches to manage anti-TKR resistance will be discussed.

## 1. Introduction

Lung cancer is responsible for most cases of cancer-related deaths [1, 2]. This pathology is a heterogeneous disease and can be histologically classified into two major different groups: small-cell lung cancer (SCLC) and non-small-cell lung cancer (NSCLC). NSCLC accounts for 85% of the primary lung carcinomas [3] and yields the highest mortality rate of malignant tumors worldwide. Within this group of NSCLC we can find several subhistologic groups, of which the most common are adenocarcinoma (ADC) and squamous cell lung cancer (SCC). The majority of patients are clinically diagnosed at advanced stages, with a 5-year survival rate of 15% [4]. For this reason, the identification of oncodrivers, novel therapeutic targets, and clinically relevant predictive or prognostic biomarkers for this disease is of high importance.

**Figure 1:**
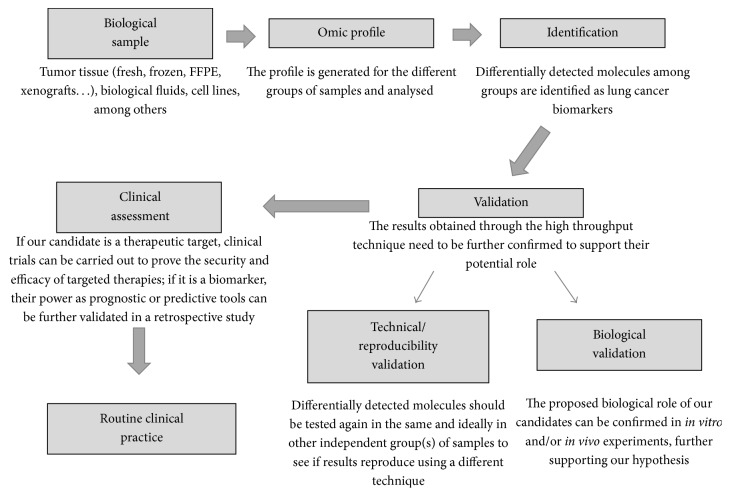
Workflow of the identification and validation of biomarkers and therapeutic targets through omics techniques.

The development of technology has made the analysis of high amounts of samples feasible through the so-called high throughput techniques. Regarding cancer, these techniques have allowed the identification of key biomarkers with translational relevance in lung cancer. Genomics, transcriptomics, miRNAomics, epigenomics, proteomics, metabolomics, lipidomics, glycomics, and many other “omics” techniques have been used to decipher the molecular pathogenesis of this disease. A proposed workflow for this aim through the use of the “omics” is shown in Figure 1. The first step would be the identification of candidate specific biomarkers, which will be differentially expressed among different experimental or clinical conditions. Different kind of biological samples, such as tumor tissue, cell lines, or biological fluids, can be used in this step. Then, the identified biomarkers must go through technical and biological validations that will confirm preliminary results. If a specific biomarker has the potential to be therapeutically targeted, clinical trials can be subsequently carried out to establish the security/efficacy of one certain drug against molecule target. Additionally, retrospective studies involving patient samples and clinical data can be carried out to support the role of biomarker.

The application of high throughput techniques in lung cancer has thus identified many gene alterations with a potential oncogenic role in this pathology. Many of these alterations take place in tyrosine kinase proteins, which integrate the so-called “kinome”. Among them, the tyrosine kinase receptors (TKRs) (Table 1) are especially relevant in this pathology. These kinds of receptors have a common molecular structure, involving three modules with a different function: the extracellular domain, able to bind the receptor ligands; the transmembrane domain, which inserts the receptor in the plasma membrane; and the intracellular domain, which is the one with the tyrosine kinase activity [12]. Under physiological conditions, tyrosine kinase receptors bind to their ligands, which produce receptor dimerization and transactivation [13]. Transactivation occurs through the phosphorylation of concrete amino acid residues in each receptor, which allows the binding and activation of effectors, directly or indirectly through scaffold proteins. There are several cancer-related signalling pathways which are activated in TKR signalling, like PI3K/AKT, RAS/MAPK, STAT, or PLC*γ*1 [14]. The activation of these downstream effectors will at the end modify different aspects of cell behaviour, like proliferation, cell survival and metabolism, cell migration, and control of cell cycle, among others [13, 15]. The activation of TKRs depends thus on ligand binding upon normal conditions, and it is regulated through different feedback mechanisms. Some examples of these are the action of phosphatases which dephosphorylate and thus deactivate the receptor [13] or mechanisms involving receptor internalization and degradation [16]. However, different molecular mechanisms cause uncontrolled TKR signalling, leading to carcinogenesis. Some examples of those are mutations, gene amplification, and overexpression inducing ligand-independent receptor dimerization, or malfunctioning of TKR signalling regulation mechanisms [17]. Along this review, we will discuss the importance of TKRs in lung cancer and their relevance in the therapeutical management of this disease.

## 2. Importance of TKRs in Lung Cancer

**Figure 2:**
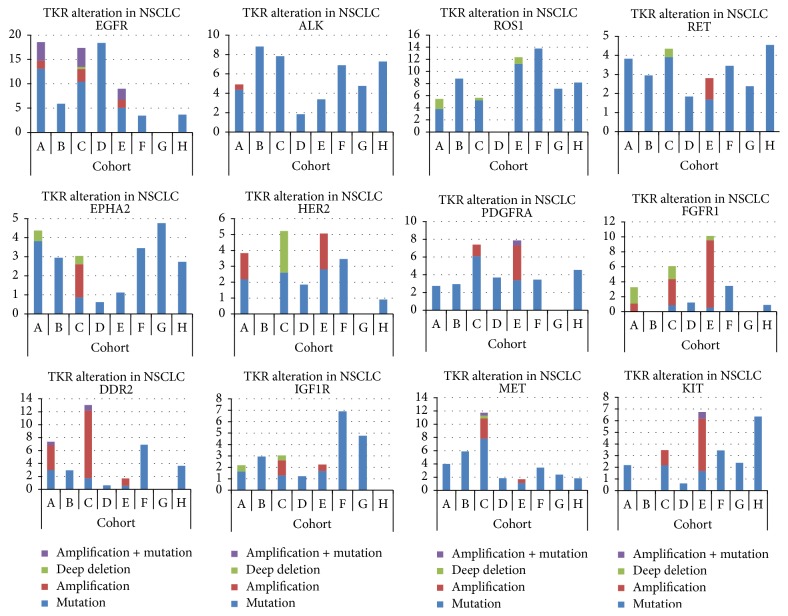
TKR alterations in lung cancer studies. Graphs showing the frequency of alterations in TKRs of relevance in the different lung cancer histologies found in different studies publicly available at http://www.cbioportal.org/. The different studies are designated by capital letters: (A) Imielinski et al., 2012 [5]; (B) MSKCC (Memorial Sloan Kettering Cancer Center) 2015; (C) TCGA, 2014 [6]; (D) Ding et al., 2008 [7]; (E) TCGA, 2012 [8]; (F) Peifer et al., 2012 [9]; (G) Rudin et al., 2012 [10]; and (H) George et al., Nature 2015 [11]. Only studies A, C, and E have information about copy number alterations. ADC: lung adenocarcinoma; SCC: lung squamous cell carcinoma; SCLC: small-cell lung cancer.

Alterations in TKRs have been detected in every histological type of lung cancer (Table 1, Figure 2), with a potential role in the development of this disease.

### 2.1. TKRs in Lung Adenocarcinoma

There are well characterized lung cancer driver oncogenes, especially in ADC. In this lung cancer histology, mutations in KRAS and EGFR and ALK translocations account for the 15–25%, 10–35%, and 2–5% of cases, respectively, the two latter being TKRs [18]. Epidermal growth factor receptor (EGFR, HER1) is part of a family of four TKRs (HER1–4) involved in the pathway of epidermal growth factor (EGF). Some identified somatic activating mutations on this gene, like deletion del19E746-A750 and the point mutation L858R, were found to be a good prognostic biomarker. These mutations have been associated with a good response to EGFR-tyrosine kinase inhibitors (TKIs). Some of these inhibitors, such as erlotinib, afatinib, and gefitinib, have been approved for clinical use mainly as first/second treatment line for EGFR-mutated adenocarcinoma patients [19–21]. The most frequent mutations detected in EGFR are located in exons 19 and 21 and are present in 45% and 41% of EGFR-mutated tumors, respectively [22]. These mutations cause the constitutive activation of the receptor resulting in uncontrolled EGFR signalling [23]. Independently from the role of EGFR in the membrane, where it activates its associated signalling pathways via ligand binding or as a cause of overexpression or mutation, EGFR is internalized to the nucleus. Once in the nucleus, EGFR is capable of acting as a coactivator for several oncogenes as Cyclin D1, nitric oxide synthase, Aurora Kinase A, c-Myc, and B-Myb [24]. Furthermore, nuclear EGFR promotes DNA replication and repair through its association to proliferating cell nuclear antigen (PCNA) [25] and DNA dependent protein kinase [26]. Interestingly, nuclear EGFR could also be involved in resistance to several cancer therapies like cetuximab, gefitinib, and even radiation and chemotherapy [27]. Other studies have focused on the potential role of EGFR as biomarker in noninvasive patient samples. Many of these have shown the feasibility and potential of EGFR mutation determination in circulating free DNA from peripheral blood samples. These studies show that there is a good correlation between tumor tissue and blood samples EGFR mutation status [28, 29]. Furthermore, circulating free DNA EGFR mutation status has been associated with clinical outcome to EGFR-TKI treatment [30, 31]. These works provide evidence that noninvasive samples can be used to detect EGFR activating mutations [32].

The second most important altered TKR in lung adenocarcinoma, ALK (Anaplastic Lymphoma Kinase), is a transmembrane TKR integrated in the insulin receptor superfamily. This gene is susceptible to suffer a rearrangement resulting in a fusion protein together with the echinoderm microtubule-like protein 4 gene (EML4), which is involved in the correct microtubule formation. This fusion protein consists of the intracellular tyrosine kinase domain of ALK and different truncations of EML4, resulting in different fusion protein variants [53, 54]. These gene rearrangements have been detected in NSCLC [55] and seem to be not mutually exclusive with EGFR and KRAS alterations [33, 49, 56]. Currently, there is a first-generation FDA approved therapy for locally advanced and metastatic NSCLC patients harbouring this rearrangement, called crizotinib [57, 58]. Furthermore, there is clinical evidence that patients treated with crizotinib show higher efficacy when compared to pemetrexed-plus-platinum chemotherapy [59]. The other most important alteration in lung cancer, KRAS mutation, does not occur in a TKR gene. However, there are studies showing the relevance of TKRs in KRAS-dependent NSCLC biology and therapy. One recent example of the involvement of TKRs in KRAS mutated lung adenocarcinoma is DDR1. DDR1 is a tyrosine kinase receptor that is activated by several types of collagen. In a recent work, DDR1 TKR was found to be overexpressed in hyperplastic tissue in a lung KRAS-mutant mouse tumor model. The genetic silencing and pharmacological inhibition of this gene impaired the tumor initiation and progression. Furthermore, in KRAS-mutant patient-derived lung xenografts treated with a combination of DDR1 and Notch signalling inhibitors, a similar efficacy as compared to standard chemotherapy was achieved [60].

Apart from these three oncodrivers, accounting for an important percentage of lung cancer cases, new molecular alterations in TKRs associated with oncogenicity have been recently described. One example of altered TKR is the ROS1 gene, which has been proved to be involved in lung cancer. This receptor belongs to the subfamily of tyrosine kinase insulin receptor genes. ROS1 fusions were detected as a potential oncodriver in a NSCLC cancer patient (with the CD74-ROS1 rearrangement) [61]. The ROS1 kinase domain in these fusion proteins is constitutively active and presents sensitivity* in vitro* to TKIs like TAE684 [62]. The analysis of the clinicopathological characteristics of a patient cohort showed that ROS1-positive patients, with an incidence of 1,7%, integrate a genetic subtype of NSCLC with similar characteristics to ALK-positive patients [63].

Another case of oncodriver TKR is RET, which is a tyrosine kinase receptor for the GNDF-family ligands (GFLs). A RET translocation (KIF5B-RET) was first identified by whole genome and transcriptome sequencing of tumor tissue from an adenocarcinoma patient in an advanced stage [64]. After that, several research groups have reported the presence of these fusions in patients who integrate a new molecular subset of lung cancer with similar characteristics to ALK-positive and ROS1-positive patients [65, 66]. Furthermore, the oncogenic potential of these fusions has been proved in NIH3T3 and Ba/F3 cells [65, 66]. Since their discovery, RET fusions have been reported in an increasing number of patients, comprising 1-2% of NSCLC patients, and they show mutual exclusivity with other known driver oncogenes [65].

Thanks to Next Generation Sequencing (NGS) and Fluorescence* In Situ* Hybridization (FISH) techniques, an oncogenic fusion involving another TKR, NTRK1, was identified in 3 ADC patients with no known oncogenic alterations in a work involving 91 ADC patients [67]. Furthermore, it has been reported that this TKR can be successfully targeted* in vitro*, as drugs like lestaurtinib, ARRY-470, and crizotinib have proved efficacy in Ba/F3 cells expressing NTRK1 fusion proteins [67].

Another TKR which has proved to be of relevance in lung ADC is ERBB2. ERBB2 (HER2) is part of the ERBB family of receptor tyrosine kinases, as EGFR. Constitutive activation of this TKR through amplification and mutation has been reported in NSCLC [68], where exon 20 insertions in this gene are common [69, 70].* In vitro* experiments show that cell lines harbouring exon 20 insertions in this gene are sensitive to trastuzumab and to the EGFR/ERBB2 dual inhibitors afatinib and neratinib [50, 71, 72].

Furthermore, many works have been carried out to identify novel oncodrivers in adenocarcinoma with the help of high throughput technologies. In a collaborative work 188 human lung adenocarcinomas DNA samples were sequenced for 623 genes with a potential role in cancer. This analysis revealed more than 1000 somatic mutations which occurred preferably in 26 genes, 30% of which were TKRs. Two of those were ERBB3 and ERBB4, from the same receptor family of ERBB1 (EGFR) and ERBB2. In ERRB4, a total of 9 mutations were detected. From those, two were located on the protein kinase domain and five around the receptor ligand binding domain. In ERBB3, 3 mutations were found in the ligand binding domain. In another tyrosine kinase receptor from the ephrin family, EPHA3, 11 mutations were found in the extracellular and kinase domains. One of those mutations found in EPHA3 kinase domain, K761N, is located at a highly conserved position analogous to the mutation K641 in FGFR2. A significant number of mutations were also identified in VEGFR and FGFR family member, especially in KDR and FGFR4, where four and three tyrosine kinase domain mutations were found, respectively [7]. In another study, 20 cases of NSCLC patients with no previously identified EGFR mutations were selected for NGS. Mutations were found in MET, FGFR3, and ERBB4 and two previously undescribed EGFR mutations were reported. Furthermore, pathogenic mutations were also reported in VEGFR2, FGFR2, and RET [51].

### 2.2. TKRs in Lung Squamous Cell Carcinoma

As discussed in the latter paragraphs, during the last years, the most actionable TKR oncogenic mutations have been described in ADC. However, research on tumor biology of SCC has not resulted in as good results as in ADC at a therapeutical level so far. For that reason, efforts are currently being carried out to identify new oncogenes involved in the development of lung tumors of this histology. One of the most interesting TKRs in lung SCC is FGFR1. FGFR1 is part of the type 4 family of TKRs and has the ability to regulate proliferation via the MAPK and PI3K pathways, similarly to EGFR. A screen of SCC samples detected focal amplifications of the FGFR1 gene [73]. This alteration is characteristic of lung SCC, with 21% cases harbouring this amplification. FGFR1 has proven to be a potential oncogenic driver* in vitro*, where FGFR1-amplified cell lines have shown dependency on FGFR1 [73–75]. However, the response rates observed in FGFR therapy in SCC are not as promising as in EGFR or ALK-directed therapy in ADC. There is increasing evidence that this may be due to the lack of correlation between FGFR1 DNA amplification and mRNA and protein expression, so that cell lines with low FGFR1 expression are insensitive to FGFR inhibitors even if they harbour FGFR1 amplification [38, 76]. FGFR-TKI-therapy is currently under development, with small molecule inhibitors like PD173074, which inhibit the growth of FGFR-amplified lung cancer cell lines and xenograft models [74, 75]. Another member of the FGFR family, FGFR2, has been identified as an interesting target in a subset of lung SCC patients. This gene is altered in 4-5% patients of NSCLC [77] and ongoing and recently completed clinical trials are going to test their potential role as therapeutical target in patients.

DDR2 is another receptor tyrosine kinase which has proven to be of relevance in SCC. This receptor binds to collagen in the extracellular matrix and regulates proliferation and migration. Mutations in DDR2 have been identified in this histologic subtype [78], suggesting a potential oncogenic role for this gene. Furthermore, an* in vitro* study has found out that reduced proliferation after DDR2 silencing or dasatinib treatment is produced in DDR2-mutant cell lines [78]. Several studies propose an incidence of DDR2 mutations of approximately 3-4% in SCC patients and although no specific anti-DDR2 therapy has been developed, ABL kinase inhibitors such as dasatinib or imatinib display activity against DDR2 [78–81]. Two studies have reported tumor shrinkage after treatment with dasatinib in SCC patients with the S768R DDR2 mutation [40, 78]. However, there is still some controversy about the role of DDR2 in tumorigenesis. This is because the DDR2 ligand, collagen, accumulates during lung tumor progression [39, 41]. However, collagen inhibits cancer cell growth through DDR2-dependent cell cycle arrest in some kinds of cancer [82, 83]. Furthermore, DDR2 mRNA levels are reduced in lung tumor as compared to matched nontumoral tissue [84]. All of this data suggests a possible context-dependent role for DDR2 in lung tumorigenesis, which needs to be further studied.

In the last years, many other TKRs are gaining attention in the study of the oncogenesis of SCC. One example is the insulin-like growth factor receptor 1 (IGF1R), which is involved in proliferation and inhibition of apoptosis [42, 46]. There is evidence of the oncogenic role of IGF1R in lung cancer, with especial relevance in SCC [47]. Several studies proposed that high-level expression of IGF-R1 is characteristic of SCC and can act as a prognostic indicator [48]. Another one is PDGFRA, a TKR from the family of the platelet-derived growth factor receptors involved in tumoral angiogenesis [85]. PDGFRA is frequently expressed in the tumor stroma, as well as in cancer cells, and its activation has been reported in 13% of NSCLC patients [61]. Alterations in this gene have been reported mainly in SCC [86]. On the other hand, a member of the Eph family of receptors,* EphA2,* was shown to be a relevance biomarker in SCC, where it promotes invasion, cell motility, and angiogenesis through the activation of* Src* [87, 88].* EphA2* mutations are rare in NSCLC but are mainly present in SCC [89].

EGFR vIII, a mutated form of EGFR found in SCC, harbours deletion in exons 2–7. This EGFR variant is not present in normal tissues and causes uncontrolled cell growth in tumors [90]. Furthermore, there is* in vivo* evidence of the oncogenic role of EGFR vIII in a NSCLC murine model and of the efficacy of an EGFR inhibitor, HKI-272, in this model [91]. Several studies have detected this EGFR variant in 2–5% of SCC patients, but not in ADC [91, 92].

### 2.3. TKRs in Small-Cell Lung Cancer

The molecular pathology of small-cell lung cancer has not yet been addressed as much as in non-small-cell lung cancer. However, some molecular alterations with the potential to be oncodrivers in this lung cancer histology have been identified. In one recent study, DNA from 98 SCLC tumors was sequenced and analysed for genomic alterations. Mutations in EGFR (5% of cases) and KIT (6%) and amplification of FGFR1 (4%), EPHA3 (3%), PDGFRA (2%), and MET (2%) were detected, suggesting that these TKRs could have a role in lung SCLC oncogenesis [11, 93]. But probably the most studied TKR in this lung cancer histology is FGFR1. This FGFR has been suggested as an oncodriver in SCLC [11, 94]. High-level expression of FGFR1 has been found in SCLC patients as compared to healthy individuals. Elevated expression is associated with advanced stage and poorer overall and recurrence-free survival [95]. In another study involving an Asiatic SCLC patient cohort, FGFR1 amplification correlated with poorer disease-free survival to first-line chemotherapy [52]. Furthermore, there is* in vitro* and* in vivo* evidence that anti-FGFR therapy is effective in FGFR1 amplified SCLC [96, 97]. Other TKRs are often overexpressed in SCLC and could have protumorigenic effects in this lung cancer subtype. IGF-1R protein levels have been reported to be high in 95% of SCLC cell lines [98, 99]. VEGFR high levels have been reported in SCLC patients and related to higher tumor stage, disease progression, chemotherapy resistance, and poorer outcome [98].

### 2.4. Other TKRs in Lung Cancer

Some TKR alterations are not specific of one concrete lung cancer histology. The TKR MET has proved to be of relevance in NSCLC after the large scale molecular profiling work by The Cancer Genome Atlas (TCGA) in lung ADC [100]. MET alterations were found in 7% of tumors and were mutually exclusive with other known oncogenes, supporting the role of MET as an oncogene. The most common alterations for this gene are overexpression, amplification, and exon 14 skipping [36, 101, 102]. In one study involving lung cancer patient samples, they found a correlation between Notch-1 and c-MET coexpression and a poorer prognosis. They also found an association between MET expression and advanced stage [37]. Currently there are many MET-targeted drugs in clinical development, such as small molecule inhibitors, molecules which prevent the binding of MET to its ligand HGF, and monoclonal antibodies [34, 103]. However although some of these drugs have demonstrated high efficacy* in vitro,* clinical trials results have been disappointing [104, 105]. Nonetheless, further trials are currently in progress, aiming to get better results by a better patient selection [106]. Apart from MET, it has been recently reported that the VEGFR receptor family could have a prognostic potential in lung cancer. In a meta-analysis covering 74 studies with a total of 7631 patients, it was reported that VEGFR1 expression is an indicator of poor prognosis in NSCLC. In this study, it was observed as well that combined high expression of VEGFR2 and VEGFA, or VEGFR3 and VEGFC, featured discrimination power as prognostic biomarkers [107]. In another study involving surgically resected NSCLC, different patterns of coexpression of HER family receptors have been associated with a shorter disease-free and overall survival [108].

## 3. Therapy Improvement through Biomarker Integration and Resistance Managing: EGFR Mutations and ALK Translocation

As commented before, acquired resistance to targeted therapy is a relevant problem in clinics. Besides, there are tumors potentially sensitive to a targeted therapy but which show innate resistance. For all these reasons, current efforts are focused on the managing and avoidance of these resistances, as well as on the improvement of eligibility criteria for TKI-therapy.

In the case of EGFR, 20–50% of patients with clinical or biological predictors of anti EGFR-therapy sensitivity do not respond [109]. This primary resistance is associated with EGFR exon 20 insertions [110]. And even if the patient responds to therapy, acquired resistance arises, due to molecular mechanisms like bypass signalling. This mechanism involves the reactivation of downstream signalling pathways via amplification of other TKRs (like MET or HER2) and mutations of downstream members of EGFR-signalling pathway (such as PIK3CA, KRAS, and BRAF) and even through ALK gene rearrangement [43, 44, 111]. Besides, to overcome sensitivity to EGFR-targeted therapies, some other tumors undergo a phenomenon similar to epithelial to mesenchymal transition (EMT), where the tumor can even suffer a change in histology, from NSCLC to SCLC [45, 112].

However, the most frequent cause of acquired EGFR-TKIs resistance, accounting for 50% of resistant cases, is a mutation in exon 20 of the EGFR gene, T790M [113]. Nonetheless, this mutation has been found as well in patients who have not received TKI-therapy [114, 115]. To overcome this resistance mechanism, second- and third-generation EGFR-TKIs have been developed and are currently under clinical trials [116]. The second-generation EGFR-TKIs, like dacomitinib, afatinib, and neratinib, display a higher affinity for the EGFR-tyrosine kinase domain [117]. They are pan-HER inhibitors and active against the T790M mutation. Unfortunately, second-generation TKIs show little activity in tumors which have acquired resistance to first-generation EGFR-TKIs [118, 119]. The third-generation EGFR-TKIs AZD9291 and rociletinib have proved efficacy against the T790M mutation* in vitro* [120] and in two Phase I-II clinical trials [121, 122]. Unfortunately, new generation EGFR-TKI would only postpone the inevitable, as new resistance mechanism will arise. In the case of AZD9291, a resistance mechanism occurring through the C797S mutation has been already identified [123].

Apart from second- and third-generation EGFR-TKIs, other treatment strategies are being developed to overcome acquired resistance. The switching to chemotherapy after resistance has appeared to be the most accepted approach, although there are several retrospective studies with inconsistent results to this respect [124, 125]. Another alternative therapy which is currently under clinical assessment is the combination of EGFR-TKIs and chemotherapy. Up to date, the results on the effectiveness of this combination therapy are not conclusive [126, 127], but ongoing clinical trials on this issue could clarify if this approach could be beneficial for patients with EGFR-TKI acquired resistance. Thanks to the identification of the molecular mechanisms leading to acquired resistance to TKIs, approaches with a more targeted design are being designed [128–132]. Many current research works bet on the combination of an EGFR-TKI with another molecularly targeted agent, for therapeutic tumor resensitization to anti-EGFR-therapy, with interesting preclinical results [133–135].

Furthermore, other more novel approaches aiming at EGFR-therapy resensitization have been recently proposed. A bispecific EGFR/MET antibody, called JNJ-61186372, has recently showed a potent inhibition of EGFR downstream effectors, resulting in tumor regression in NSCLC xenografts [136]. In another recent work, cetuximab delivery through a mesoporous silica nanoparticle (MP-SiO2 NP) suppressed progression of EGFR-therapy-resistant xenografts [137].

On the other hand, the expression of several lncRNAs has been associated with EGFR-targeted therapy resistance, suggesting a potential role for them as predictive and therapeutic biomarkers [138]. In addition, Park et al. found that a low EGFR/MET ratio was also predictive of poor response to anti-EGFR-therapy [139]. Another recent research work involving patients receiving erlotinib therapy has identified TGF-*α* and high soluble EGFR serum levels as negative and positive response predictive biomarkers to erlotinib, respectively [140].

Furthermore, the evaluation of circulating free DNA from liquid biopsies as a noninvasive method for resistance monitoring is currently under development, and promising results have been obtained for the detection of the T790M, c-MET amplification, and the C797S mutation [31, 141].

For ALK gene rearrangement targeted treatment, as in the case of EGFR-TKI treatment, acquired resistance arises in less than a year after the beginning of the treatment [142]. The best documented acquired resistance mechanisms to crizotinib-based therapy are mutations in the ALK gene [143–145]. These mutations represent the 28% of crizotinib-resistant cases. Some of them take place in the ATP-binding pocket of ALK and others occur distant to the ATP-binding site, but all of them finally reduce the ALK affinity for crizotinib [146]. Many other mechanisms of acquired resistance have been described for this therapy. One of those consists in the amplification of the ALK gene [146], which has been reported in the 18% of patients treated with crizotinib. Another mechanisms of reported crizotinib acquired resistance are KRAS mutations, amplification of KIT, and increased phosphorylation of EGFR [144, 146, 147]. Recently, it has been reported that NSCLC cells can acquire resistance to anti-ALK therapy through the activation of other receptor tyrosine kinases. In this work, two NSCLC cell lines with the ALK translocation were treated with alectinib, a potent and selective ALK inhibitor, and resistant clones were established. In one of these cell lines, the translocation was lost and increased activation of IGF-1R and HER3 was detected, and when these two signalling pathways were inhibited, cells were resensitized against alectinib. The second alectinib resistant cell line showed MET activation [148]. To overcome these resistance mechanisms, a second-generation ALK inhibitor, ceritinib, has been developed. Ceritinib has been recently approved for patients with acquired resistance or intolerance to crizotinib [149]. However, ceritinib is only active against some of the ALK mutations [144, 146]. Furthermore, a new ALK-targeted drug, alectinib, showed higher potency than crizotinib. This drug was approved in Japan for treatment of recurrent ALK rearrangement NSCLC patients [150].

On the other hand, noninvasive detection of ALK rearrangements has proven to be feasible. In one work, the EML4-ALK translocation has been detected in circulating blood platelets. This is because platelets are able to sequester RNA released into the blood by tumor cells, and this is why the ALK translocation could be found in platelet RNA transcripts [151].

Currently, different novel therapeutic agents with improved characteristics are under evaluation [152] and, as in the case of EGFR, combination therapy approaches are gaining increasing interest in overcoming resistance [57, 153–155]. Again, some efforts are currently being made to understand the molecular biology of ALK therapy resistance, similarly to EGFR [156]. The better understanding of the molecular mechanisms underlying the sensitivity to or ineffectiveness of this therapy will help in the identification of novel predictive biomarkers and even new targets to address.

## 4. Research on Novel Targets

The recent discovery of genetic alterations on TKRs in patient samples has opened the door to research works aiming to find an appropriate and targeted therapy for subsets of patients with characterized oncogenic alterations.

For ROS1-fusion genes,* in vivo* models have been generated to test the efficacy of ROS1-targeted agents [157, 158]. Two transgenic mouse models have been produced, in which overexpression of CD74-ROS1 or SDC4-ROS1 fusion variants takes place in lung alveolar type II cells. In these transgenic models it was shown that these translocations have oncogenic potential* per se*, and that crizotinib and ASP3026 (an ALK/ROS1 inhibitor) are potentially efficacious therapies to target them [157]. Furthermore, acquired resistance mechanisms have already been identified for crizotinib in ROS1-rearranged patients, like the G2032R mutation [159]. Some TKIs, as cabozantinib and foretinib, seem to be effective against this resistance mutation [146, 160].

RET rearrangements have also been object of interest to study potential targeted therapies in lung adenocarcinoma. As in the case of ROS1, genetically engineered mouse models have been established. In one of these models, the KIF5B-RET fusion was exogenously expressed specifically in lung alveolar epithelial cells, generating multiple tumors in the lungs. In this model, vandetanib, a RET inhibitor approved to be used in thyroid carcinoma, showed antitumor efficacy [161]. This drug is currently under clinical assessment in a phase 2 trial involving NSCLC patients. The potential applicability of some other TKIs has been assessed in preclinical models with appealing results. Some examples are sunitinib and sorafenib, currently in clinical trials [65, 66]. Another example is dovitinib, which has shown* in vitro* and* in vivo* antitumor efficacy in a work involving a cell line harbouring the CCDC6-RET fusion variant and its xenografts. In this work, a mechanism of resistance to dovitinib through the activation of Src was also described, and the use of a Src inhibitor, saracatinib, was proposed to overcome this resistance [162].

In lung SCC, probably the TKR which has attracted the most attention is FGFR1. Regarding anti-FGFR therapy, there has been some controversy about predicting treatment effectiveness. There are some works in which* in vitro* and* in vivo* xenograft models have showed a correlation between efficacy of anti-FGFR therapy and FGFR1 amplification [73, 163]. However, a more recent work has proven that FGFR-TKI sensitivity depends on FGFR1 mRNA or protein expression levels and not on FGFR1 gene amplification [38]. Another member of the FGFR family, FGFR2, has gained interest in this histological subtype. Some preclinical models of FGFR2-driven lung SCC have been established. In one of those, a genetically engineered mouse model expressing a mutated variant of this gene proved to be oncogenic in a p53 deficient background. Furthermore, these FGFR2 mutant tumors were sensitive to FGFR inhibition [77]. Another TKR in which a lot of preclinical work has been developed is DDR2. DDR2 mutation has been associated with clinical response to dasatinib in SCC [40]. In addition, two acquired resistance mechanisms to dasatinib have already been described* in vitro*, that is, the DDR2 T654I mutation and NF1 loss [164]. Currently, novel DDR2 inhibitors with a higher selectivity are under development.

Regarding SCLC, several studies have investigated the role of FGFR1 in preclinical models [96, 97]. The FGFR inhibitor PD173074 appears to inhibit cell growth in several FGFR1-overexpressing cell lines and in cell line xenograft models, comparably to cisplatin treatment [97]. Currently, there are several FGFR TKIs under clinical assessment, some of which are more selective, like AZD4547 or BGJ398, and some of which are more promiscuous, like JNJ-42756493. However, preliminary results from clinical trials have not been very successful.

Certainly, the targeted therapy has shown promising results, but so far the appearance of resistances seems unavoidable. For this reason, many current efforts are focused on therapeutic approaches that delay the appearance of resistances. Ongoing work is assessing the effectiveness of many other options of combination, including a TKI against the same target, but with a different resistance profile [126, 165], and combination with immunotherapy [166], among others.

## 5. Directions of Future Research and Conclusions

Thanks to the omics techniques and their high throughput capacity of analysis, many alterations with potential involvement in lung cancer have been identified and validated in the last years, leading to improvements in clinical practice. Many of these aberrations occur in TKRs, inducing a deregulated downstream signalling that leads to tumorigenesis. Due to the functional nature of TKRs, their action can be pharmacologically inhibited, making the TKRs very appealing for research in cancer. Indeed, addressing the TKRs has made very interesting achievements in lung cancer treatment, resulting in the development of targeted therapies that have provided a substantial benefit for patients eligible for those therapies. However, the benefit derived from any targeted therapy is unfortunately transient, due to the development of resistance to these therapies. Current and future research efforts will be focused on understanding the molecular nature of these resistances, aiming to find novel predictive biomarkers of therapy response and new therapeutic approaches that prevent, or at least delay, the appearance of resistances and tumor regression.

## Figures and Tables

**Table 1:** Summary of prevalence of TKR molecular alterations, divided by the main lung cancer histologies, and examples of TKIs available for every alteration.

Alteration	NSCLC		SCLC	TKI available
	ADC	SCC		
EGFR mutation	10–15% [33]	5% [34]	<5% [35]	Erlotinib, Afatinib, Gefitinib, AZD9291, rociletinib
EGFR vIII mutation	Very rare [36, 37]	—	5% [36, 37]	HKI-272
HER2 overexpression	5–9% [38]	—	3–5% [38]	Afatinib, Neratinib, Trastuzumab
HER2 mutation	2% [38]	—	1% [38]	
HER2 amplification	0,9% [38]	—	—	
FGFR1 amplification	1–3% [39]	4–6% [34]	20% [40, 41]	BGJ398, AZD4547, JNJ-42756493
FGFR rearrangement	Very rare [42]	—	1% [42]	
MET amplification	3–21% [43–45]	2% [43–45]	3–21% [43–45]	Crizotinib, Tivantinib
MET mutation	2% [38]	—	1% [38]	
DDR2 mutation	1% [46–48]	—	4% [46–48]	Dasatinib
ALK rearrangement	2–7% [33]	—	1% [49]	Crizotinib, alectinib, Ceritinib
ROS1 rearrangement	1,7% [50]	—	—	TAE684
RET rearrangement	0,9% [7, 51]	—	—	Vandetanib, ASP3026, Cabozantinib, Foretinib
KIT mutation	—	6% [34]	—	Axitinib, Imatinib
PDGFRA amplification	3.8% [52]	2% [34]	8.7% [32]	Crenolanib

NSCLC: non-small-cell lung cancer; ADC: adenocarcinoma; SCC: squamous cell carcinoma; SCLC: small-cell lung cancer.
