# Corrigendum to “Tyrosine Kinase Receptor Landscape in Lung Cancer: Therapeutical Implications”

**DOI:** 10.1155/2018/3714684

**Published:** 2018-07-19

**Authors:** A. Quintanal-Villalonga, Luis Paz-Ares, Irene Ferrer, S. Molina-Pinelo

**Affiliations:** ^1^Medical Oncology Department, Hospital Universitario Doce de Octubre, 28041 Madrid, Spain; ^2^Centro Nacional de Investigaciones Oncológicas (CNIO), 28029 Madrid, Spain; ^3^University of Seville, Seville, Spain

In the article titled “Tyrosine Kinase Receptor Landscape in Lung Cancer: Therapeutical Implications” [1], there was a missing affiliation for the second author. The corrected authors' list and affiliations are shown above.
